# Reaction norm models for the evaluation of reproductive and productive performance in Nellore cattle

**DOI:** 10.1007/s11250-026-04947-5

**Published:** 2026-03-24

**Authors:** F. L. Menezes, S. I. Araújo, R. B. Lobo, L. R. M. Nakabashi, F. Baldi, N. L. Pavan, G. C.I Seidel, C. V. Araújo

**Affiliations:** 1 Núcleo de Pesquisa e Ensino em Melhoramento Animal- NUPEMA, Universidade Federal de Mato Grosso, Sinop, Mato Grosso 78557-267 Brazil; 2 Universidade Federal de Mato Grosso – UFMT, Sinop, MT Brazil; 3 ANCP- Associação Nacional de Criadores e Pesquisadores, Ribeirão Preto, São Paulo, 14020-230 Brazil

**Keywords:** Animal breeding, Animal performance, Beef cattle, Genetic evaluation

## Abstract

This study evaluated genotype × environment interactions (G×E) in Nellore cattle for growth, reproductive, and carcass traits using reaction norm models. We analyzed the records of body weight and scrotal circumference at 12 and 18 months. (W365, W450, SC365, SC450), as well as loin eye area (LEA) and subcutaneous fat thickness (SFT), from herds across different Brazilian states were analyzed. A hierarchical one-step reaction norm model was applied, including fixed effects of contemporary groups (herd, year and season of birth, and sex), cow age at calving as a covariate, and random effects for additive genetics, permanent environment, and residuals. Bayesian inference with Gibbs sampling was used to estimate posterior distributions.Sires were classified according to environmental sensitivity into four categories: extremely robust, robust, plastic, and extremely plastic. For most traits, over 90% of sires were classified as robust or extremely robust, indicating stability of ranking across environments. Exceptions were observed for SFT and SC365, where lower correlations between extreme environmental gradients suggested G×E effects. Heritability estimates ranged from 0.07 to 0.32 for body weights and reached up to 0.76 for fat thickness, with higher values observed under more favorable environmental conditions. .These findings underscore the utility of reaction norm models for identifying environmentally robust sires under the variability of tropical production systems, thereby supporting more accurate and efficient selection decisions in genetic improvement programs.

## Introduction

Maximizing productivity while maintaining sustainability is essential in beef cattle production systems, where growth and reproductive efficiency are key determinants of profitability. Growth and carcass traits are prioritized in herd selection, whereas in males, reproductive traits, particularly scrotal circumference (SC) at 365 or 450 days of age, are widely used due to their ease of measurement, low cost, and correlation with puberty onset and age at first calving (Carvalho Filho et al. 2022).

However, the expression and response of these selection traits can vary across environments due to genotype × environment interaction (G×E), which can modify genetic and environmental variances and compromise the efficiency of genetic improvement programs, particularly in countries characterized by high environmental heterogeneity (Mattar et al. 2011).

Among the available approaches, reaction norm (RN) models have been widely applied to study G×E for growth and carcass traits, describing phenotypes across different environments. (Souza et al. 2016; Ambrosini 2016; Cardoso et al. 2015; De Jong 1995). Each animal is assigned random regression coefficients, intercept (a) and slope (b), predicting the genetic value (û) as a function of the environmental gradient (û = a + bX) (Souza 2014; Rodrigues 2012). These models also allow estimation of variance components with relatively few parameters using covariance functions.

The aim of this study was to evaluate G×E effects for productive and reproductive traits in Nellore cattle using RN models and to classify sires according to the sensitivity of their genetic values across environmental gradients.

## Materials and methods

Data from Nelore cattle participating in the Associação Nacional de Criadores (ANCP) breeding program, across herds in different Brazilian states, were used. Records included body weights and standardized scrotal circumferences at 12 and 18 months of age (W365, W450, SC365, SC450), as well as loin eye area (LEA) and subcutaneous fat thickness (SFT).

Animals’ birth months were grouped into two seasons corresponding to periods of low (April–September) and high (October–March) rainfall, and these groups were used to define contemporary groups, including herd, year and season of birth, and sex, to control for systematic environmental and management effects based on the data distribution. Only contemporary groups with > 4 individuals and sires with ≥ 3 progeny were retained to ensure adequate genetic connectivity. Cow age at calving was included as a covariate with linear and quadratic effects (mean ± SD = 72.63 ± 36.65 months).

A hierarchical one-step reaction norm model (Su et al. 2006) was applied to estimate the additive genetic covariance function and random effects. The model was specified as: **y**_**ijm**_**= x**_**i**_**β + £**_**j**_**+ a**_**i**_**+ b**_**i**_ £_**j**_**+ pm + ε**_**ijm**_ where: y_ijm_ is the phenotype of animal i in environment j; β: vector of fixed effects (linear and quadratic for the age of the cow); x_i_ : vector of incidence; £_j_ : random environmental effect (contemporary group); a_i_: additive genetic value of animal i; b_i_ is the regression coefficient associated with the reaction norm, representing the animal’s sensitivity to environmental changes, pm is the random effect of maternal permanent environment and ε_ijm_ represents the residual error.

Variance components and random effects were estimated using Bayesian inference with the Gibbs sampler. Markov chains of 200,000 iterations were run, with a burn-in of 20,000 cycles and thinning every 10 cycles. Convergence and chain stability were evaluated using Geweke’s diagnostic (significance level = 0.05).

Environmental gradients were defined based on ascending contemporary group solutions. Five levels were created, each representing 20% of the distribution, with the first and fifth levels corresponding to the environments with the lowest and highest phenotypic values, respectively. Additive genetic variances along the gradient were estimated using the covariance function: $\:{\sigma\:}_{{a}_{\left(i\right)}}^{2}=\:{\sigma\:}_{i}^{2}+\:{X}_{i}^{2}{\sigma\:}_{l}^{2}+2{X}_{i}{\sigma\:}_{il}$, where $\:{\sigma\:}_{i}^{2}$, $\:{\sigma\:}_{l}^{2}$ and $\:{\sigma\:}_{il}\:$are the intercept, slope, and covariance components, respectively.

Sires were classified based on the posterior mean of their reaction norm slopes (bi) into four categories: Extremely Robust (ER; within 1 SD), Robust (R; >1 to < 2 SD), Plastic (P; ≥2 to < 3 SD), and Extremely Plastic (EP; ≥3 SD), reflecting the sensitivity of their genetic values to changes in environmental gradients.

## Results and discussion

Estimates of the means with their respective standard deviations for all the traits analyzed, as well as the number of records per contemporary group and sire are presented in Table 1.

**Table 1:** Number of records (N), mean, standard deviations (SD), number of records per sire (NS) and per contemporary group (NGC) for the traits W450, W365, SC450, SC365, SFT and LEA

Trait	*N*	Mean	SD	NS	NGC
W450 ^a^	62,317	299.15	51.4	1692	1164
W365 ^b^	59,122	255.47	37.54	1667	1132
SC450^c^	23,692	24.61	3.47	931	431
SC365^d^	19,991	21.49	2.59	851	414
SFT^e^	60,535	2.74	1.38	1674	1154
LEA^f^	57,409	51.53	9.42	1648	1124

a=Weight at 450 days of age and b=Weight at 365 days of age, in kilograms; c=Scrotal circumference at 450 days of age, d=Scrotal circumference at 365 days of age, in centimeters, e =Subcutaneous fat thickness and f =Loin eye area, in centimeters

To assess the adequacy of the Markov chain lengths, the estimates obtained from the Geweke diagnostic test indicated satisfactory convergence and stationarity for all posterior means of variance components (*P* > 0.05). Therefore, the chain lengths were considered sufficient to generate reliable posterior distribution estimates.

The phenotypic means, posterior mean heritability estimates, and mean contemporary group solutions for each environmental gradient and trait are presented in Table 2. In general, posterior heritability estimates increased from the lowest to the highest environmental gradient, suggesting that animals allocated to gradients with higher contemporary group solutions, representing herds with superior production performance, also exhibited greater additive genetic variability. Consequently, genetic selection is expected to be more accurate in higher environmental gradients.

Posterior heritability estimates for W365, W450, and LEA were of comparable magnitude across gradients, ranging from 0.077 to 0.295 for W365, 0.077 to 0.277 for W450, and 0.074 to 0.324 for LEA. In contrast, SFT exhibited a much larger range across environmental gradients, increasing from 0.011 in the lowest gradient to 0.769 in the highest, reflecting greater variability in fat deposition. Estimates from intermediate gradients were consistent with those reported by Londoño-Gil et al. (2022) and Bessa (2021), who observed heritabilities ranging from 0.19 to 0.24, suggesting a possible overestimation of the heritability value of 0.769.

The observed increase in posterior mean heritability estimates across gradients, a pattern consistent for all traits, suggests that the expression of a given trait responds differently to environmental conditions. Herds with higher phenotypic performance appear to contain animals with greater additive genetic variability. Consequently, in genetic evaluations that do not account for such environmental heterogeneity, sires with progeny allocated to higher-performing herds may exert disproportionate influence on the estimation of variance components, resulting in heterogeneity of additive genetic variance.

In previous studies, Oliveira et al. (2018) reported heritability estimates for yearling weight ranging from 0.35 to 0.49 using reaction norm models. Similarly, Lemos et al. (2015), evaluating scrotal circumference and yearling body weight, reported estimates from 0.13 to 0.72 for yearling weight and from 0.32 to 0.51 for scrotal circumference. Santos et al. (2012) found higher heritabilities for body weight in Guzerá cattle using random regression models (0.47–0.70), while Barbosa et al. (2017) reported lower estimates for body weight in Nellore cattle using reaction norm models (0.19–0.26). Silva et al. (2017), evaluating W365, W450, LEA, and SC365/SC450, observed posterior mean heritabilities very similar to those obtained in the present study.

Considering other beef breeds and carcass traits, Zuim (2021) reported heritability estimates of 0.23 to 0.27 for LEA and 0.07 to 0.16 for SFT, comparable to the corresponding averages observed here. In addition to additive genetic effects, non-additive factors such as genetic group, age, sex class, and nutritional level also influence tissue deposition patterns and overall body composition in beef cattle (Coleman et al. 1993).

The development of different animal tissues exhibits allometric characteristics. According to Owens et al. (1995), adipose tissue synthesis is inversely related to muscle tissue synthesis; consequently, post-pubertal growth is characterized by limited muscle development, with weight gain primarily composed of adipose tissue. Adipose tissue is therefore the last to be deposited. As noted by Doreau and Chilliard (1997), fat deposition occurs through incorporation of pre-formed fatty acids transported by plasma lipoproteins and de novo fatty acid synthesis within adipose tissue. Animals grazing on pasture typically exhibit lower fat deposition compared to those consuming high-energy diets, such as grain-based rations.

Considering that the animals in this study were maintained on a pasture diet, which contributes to slower growth, and given the age at which they were evaluated for SFT, substantial subcutaneous fat deposition had not yet occurred, as reflected in the low mean SFT values. Similar to other traits, greater additive genetic variability was observed in the higher environmental gradients (4 and 5). In these environments, animals with earlier growth, as indicated by their body weights, are likely to display precocious finishing. Compared with the later-developing animals predominant in lower gradients, these early-growing animals increase the range of SFT values, resulting in greater variability and explaining the apparent overestimation of heritability in the upper gradients.

Physiological precocity is positively associated with both sexual and carcass finishing precocity, which explains the higher posterior heritability estimates observed for SC365 and SFT in the highest environmental gradient, as this gradient also includes animals with greater body weights.

**Table 2:** Posterior mean heritability estimates (h²) and standard deviations (SD) for W450, W365, SC450, SC365, SFT, and LEA across environmental gradients (i). W450 = weight at 450 days of age (kg); W365 = weight at 365 days of age (kg); SC450 = scrotal circumference at 450 days of age (cm); SC365 = scrotal circumference at 365 days of age (cm); SFT = subcutaneous fat thickness (mm); LEA = loin eye area (cm²)

Gradient	W450		W365		SC450	
	Mean	SD	Mean	SD	Mean	SD
1	0.077	0.005	0.079	0.005	0.156	0.014
2	0.111	0.005	0.126	0.006	0.247	0.016
3	0.144	0.006	0.169	0.007	0.301	0.017
4	0.191	0.008	0.217	0.009	0.349	0.017
5	0.277	0.010	0.295	0.011	0.425	0.018

Gradient	SC365		SFT		LEA	
	Mean	SD	Mean	SD	Mean	SD
1	0.130	0.014	0.011	0.003	0.074	0.005
2	0.238	0.018	0.152	0.014	0.126	0.006
3	0.315	0.019	0.349	0.016	0.168	0.007
4	0.385	0.020	0.557	0.015	0.221	0.009
5	0.500	0.020	0.769	0.010	0.324	0.012

W450 = Weight at 450 days of age and W365 = Weight at 365 days of age. in kilograms; SC450 = Scrotal circumference at 450 days of age and SC365 = Scrotal circumference at 365 days of age. in centimeters; SFT =Subcutaneous fat thickness. in millimeters and LEA =Loin eye area. in square centimeters

**Fig. 1:**
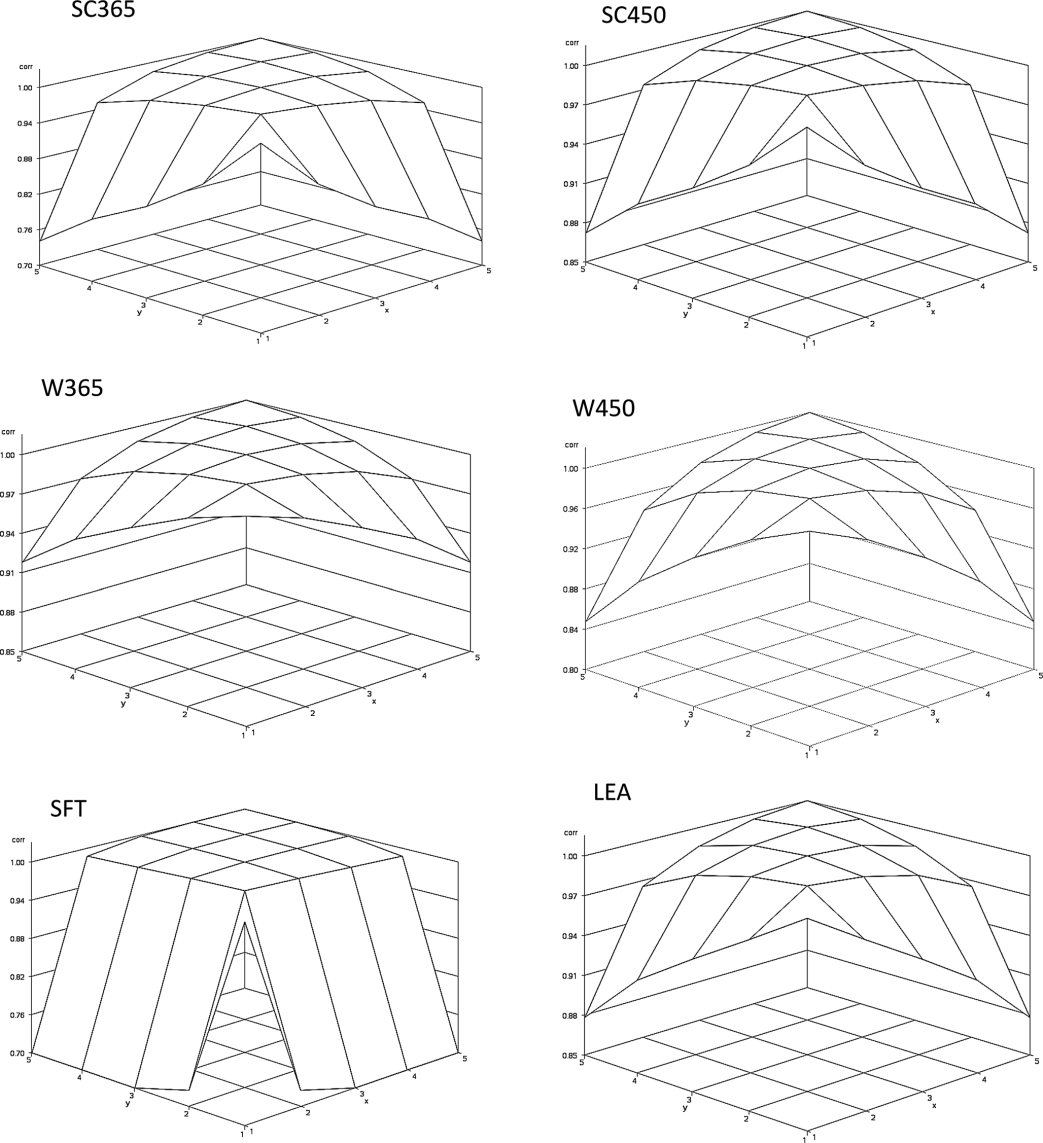
Additive genetic correlations between environmental gradients (x and y) for scrota. l circumference at 365 (SC365) and 450 (SC450) days of age, body weight at 365 (W365) and 450 (W450) days of age, subcutaneous fat thickness (SFT), and loin eye area (LEA) in Nellore cattle

Evaluation of the posterior mean estimates of genetic correlations between environmental gradients indicated lower correlations between the most extreme gradients, with higher correlations observed between adjacent gradients. Overall, correlations were high, exceeding 0.80 for most traits, except for SC365, which showed a correlation of 0.74 between the lowest and highest gradients, and particularly SFT, which exhibited the lowest posterior mean genetic correlations, ranging from 0.28 (between the first and fifth gradient) to 0.61 (between the first and second gradient; Fig. 1).

Similar patterns were observed for the posterior mean estimates of phenotypic correlations, with the largest discrepancies occurring between the extreme environmental gradients (gradients 1 and 5) and intermediate values for the remaining gradients (Fig. 1).

Considering both the posterior mean heritabilities and the genetic correlations for SC365 and especially SFT, changes in the ranking of sires are expected when evaluated across the most extreme environmental gradients. In this context, sires with the highest number of progeny in herds with superior production may have their predicted genetic values overestimated, whereas the opposite is expected for sires in herds with lower production performance.

Oliveira et al. (2018) reported high genetic correlations between adjacent environmental gradients and lower correlations between the most extreme gradients, a pattern similar to that observed in the present study, indicating that genotypes express differently across contrasting environments. Similarly, Lemos et al. (2015) reported genetic correlations for yearling scrotal circumference that were consistent with the current findings, with high correlations between nearby environments ranging from 0.71 to 1.0.

**Fig. 2:**
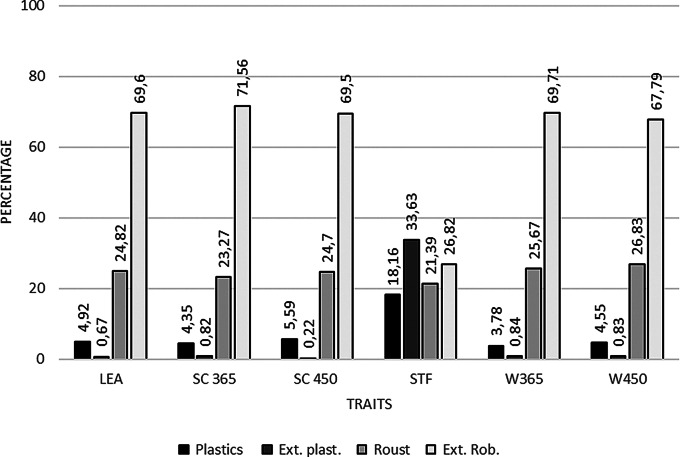
Percentage of animals classified according to environmental sensitivity classes for the traits: W450 = weight at 450 days of age (kg); W365 = weight at 365 days of age (kg); SC450 = scrotal circumference at 450 days of age (cm); SC365 = scrotal circumference at 365 days of age (cm); SFT = subcutaneous fat thickness (mm); LEA = loin eye area (cm²)

To classify the sires. the additive genetic values of each sire in each environmental Environmental gradients were predicted and sires were subsequently classified according to their environmental sensitivity as robust, extremely robust, plastic, or extremely plastic. These results indicate an overall pattern of robustness (Appendix I). Once the sires were classified by environmental sensitivity, they were sorted and grouped to estimate the percentile of each class for all traits (Fig. 2).

With the exception of SFT, more than 90% of sires were classified as robust or extremely robust, suggesting that the ranking of these animals is unlikely to change across different environmental gradients. In contrast, for SFT, approximately 52% of sires were classified as plastic or extremely plastic, likely due to the low genetic correlation between the most extreme environmental gradients. In this case, sires classified in environments with lower SFT expression differ from those in environments with higher SFT expression, reflecting genotype-by-environment interactions.

In a study by Carvalho Filho et al. (2022), evaluating the plasticity of Nellore sires for yearling body weight and scrotal circumference at yearling, most sires were classified as robust, consistent with the patterns observed in the present study for most traits. The exception was SFT, in which all classes of environmental sensitivity were represented approximately equally among the sires.

## Conclusions

Reaction norm models can serve as a valuable tool in genetic evaluations to improve the accuracy of estimated breeding values across diverse environmental conditions, thereby minimizing errors in animal classification. For all traits evaluated, with the exception of SFT, the reaction norm approach allowed identification of sire robustness, indicating that genotype-by-environment interactions had minimal effects on these traits.

The integration of reaction norm models into Nelore breeding programs can optimize the selection of sires under conditions of environmental and management diversity, thereby enhancing herd resilience and adaptability.

## Appendix I

Classification of environmental sensitivity for the genetic values of Nellore breed sires for the traits LEA, SC365, W365, SFT, W450 and SC450

**Table:** 

	LEA				SFT				SC365			
GA	P		EP		P		EP		P		EP	
	EBV	SD	EBV	SD	EBV	SD	EBV	SD	EBV	SD	EBV	SD
1	1.638	4.288	0.329	6.657	0.016	0.110	0.019	0.128	0.560	0.998	0.093	1.415
2	2.238	5.937	0.339	9.395	0.049	0.254	0.058	0.462	0.910	1.653	-0.068	2.416
3	2.624	7.003	0.346	11.158	0.083	0.425	0.096	0.820	1.104	2.021	-0.156	2.998
4	3.072	8.243	0.354	13.206	0.121	0.627	0.141	1.237	1.277	2.351	-0.236	3.524
5	3.894	10.522	0.368	16.966	0.186	0.975	0.217	1.948	1.582	2.936	-0.376	4.463
	R		ER		R		ER		R		ER	
1	0.843	2.599	0.193	1.163	0.010	0.087	0.005	0.075	0.112	0.668	0.019	0.359
2	1.19	3.582	0.242	1.52	0.026	0.157	0.005	0.089	0.214	1.063	0.024	0.479
3	1.413	4.221	0.273	1.759	0.041	0.256	0.006	0.116	0.270	1.291	0.027	0.552
4	1.672	4.968	0.31	2.041	0.058	0.378	0.007	0.155	0.321	1.497	0.029	0.621
5	2.148	6.343	0.377	2.567	0.088	0.591	0.009	0.228	0.410	1.863	0.034	0.746
	W365				W450				SC450			
GA	P		EP		P		EP		P		EP	
	EBV	SD	EBV	SD	EBV	SD	EBV	SD	EBV	SD	EBV	SD
1	3.112	17.666	-16.152	23.109	6.960	22.267	2.256	32.881	1.096	1.512	0.088	3.008
2	4.091	23.414	-21.612	30.868	9.849	28.926	3.535	43.606	1.526	2.113	0.077	4.355
3	4.841	27.822	-25.797	36.817	2.089	34.145	4.526	51.973	1.742	2.417	0.072	5.031
4	5.621	32.409	-30.151	43.007	4.789	40.475	5.722	62.095	1.931	2.684	0.067	5.623
5	6.854	39.661	-37.031	52.790	9.275	51.040	7.709	78.952	2.235	3.113	0.059	6.574
	R		ER		R		ER		R		ER	
1	3.019	10.719	0.774	4.420	3.470	14.417	1.630	7.128	0.220	1.143	-0.004	0.512
2	3.929	14.064	0.964	5.698	4.801	18.288	1.933	8.448	0.313	1.564	-0.012	0.648
3	4.626	16.637	1.109	6.691	5.834	21.360	2.167	9.534	0.359	1.777	-0.016	0.720
4	5.352	19.318	1.260	7.732	7.079	25.113	2.450	10.891	0.400	1.965	-0.020	0.784
5	6.499	23.561	1.500	9.385	9.146	31.413	2.920	13.217	0.466	2.268	-0.025	0.888

P=Plastic; EP= Extremely plastic; R= Robust; ER= Extremely robust; W450 = Weight at 450 days of age. W365 = Weight at 365 days of age. SC450 = Scrotal circumference at 450 days of age. SC365 = Scrotal circumference at 365 days of age. SFT=Subcutaneous fat thickness; LEA=Loin eye area

## Data Availability

Data will bem ade available on reasonable request.
